# *Staphylococcus aureus* biofilms: recent developments in biofilm dispersal

**DOI:** 10.3389/fcimb.2014.00178

**Published:** 2014-12-23

**Authors:** Jessica L. Lister, Alexander R. Horswill

**Affiliations:** Department of Microbiology, Roy J. and Lucille A. Carver College of Medicine, University of IowaIowa City, IA, USA

**Keywords:** biofilm, dispersal, protease, nuclease, stringent response, *Staphylococcus aureus*

## Abstract

*Staphylococcus aureus* is a major cause of nosocomial and community-acquired infections and represents a significant burden on the healthcare system. *S. aureus* attachment to medical implants and host tissue, and the establishment of a mature biofilm, play an important role in the persistence of chronic infections. The formation of a biofilm, and encasement of cells in a polymer-based matrix, decreases the susceptibility to antimicrobials and immune defenses, making these infections difficult to eradicate. During infection, dispersal of cells from the biofilm can result in spread to secondary sites and worsening of the infection. In this review, we discuss the current understanding of the pathways behind biofilm dispersal in *S. aureus*, with a focus on enzymatic and newly described broad-spectrum dispersal mechanisms. Additionally, we explore potential applications of dispersal in the treatment of biofilm-mediated infections.

## *Staphylococcus aureus* biofilms and infection

*Staphylococcus aureus* is a Gram-positive human commensal that persistently colonizes the anterior nares of approximately 20–25% of the healthy adult population, while as many as 60% are intermittently colonized (Eriksen et al., 1995; Hu et al., 1995; Kluytmans et al., 1997; Ellis et al., 2014). Studies have linked *S. aureus* nasal colonization to an increased risk of infection (Dall'Antonia et al., 2005; Ellis et al., 2014). As evidence, 65% of people with *S. aureus* infections are colonized with the same strain, whereas the percentage jumps to 80% in nosocomial infections (Weinstein, 1959; von Eiff et al., 2001; Wertheim et al., 2004). The infections that result are quite diverse, and can include acute infections, such as bacteremia and skin abscesses, that are generally caused by planktonic cells through the production of secreted toxins and exo-enzymes (Gordon and Lowy, 2008). In contrast, chronic infections are associated with a biofilm mode of growth where *S. aureus* can attach and persist on host tissues, such as bone and heart valves, to cause osteomyelitis and endocarditis respectively, or on implanted materials, such as catheters, prosthetic joints, and pace makers (Parsek and Singh, 2003; Kiedrowski and Horswill, 2011; Barrett and Atkins, 2014; Chatterjee et al., 2014). Implanted materials become coated with host proteins upon insertion, and the matrix-binding proteins on the surface of *S. aureus* facilitate attachment to these proteins and development of a biofilm (Cheung and Fischetti, 1990; Francois et al., 1996). In cases of infected medical devices, removal of the device is often necessary to treat the infection (Darouiche, 2004).

**Figure 1:**
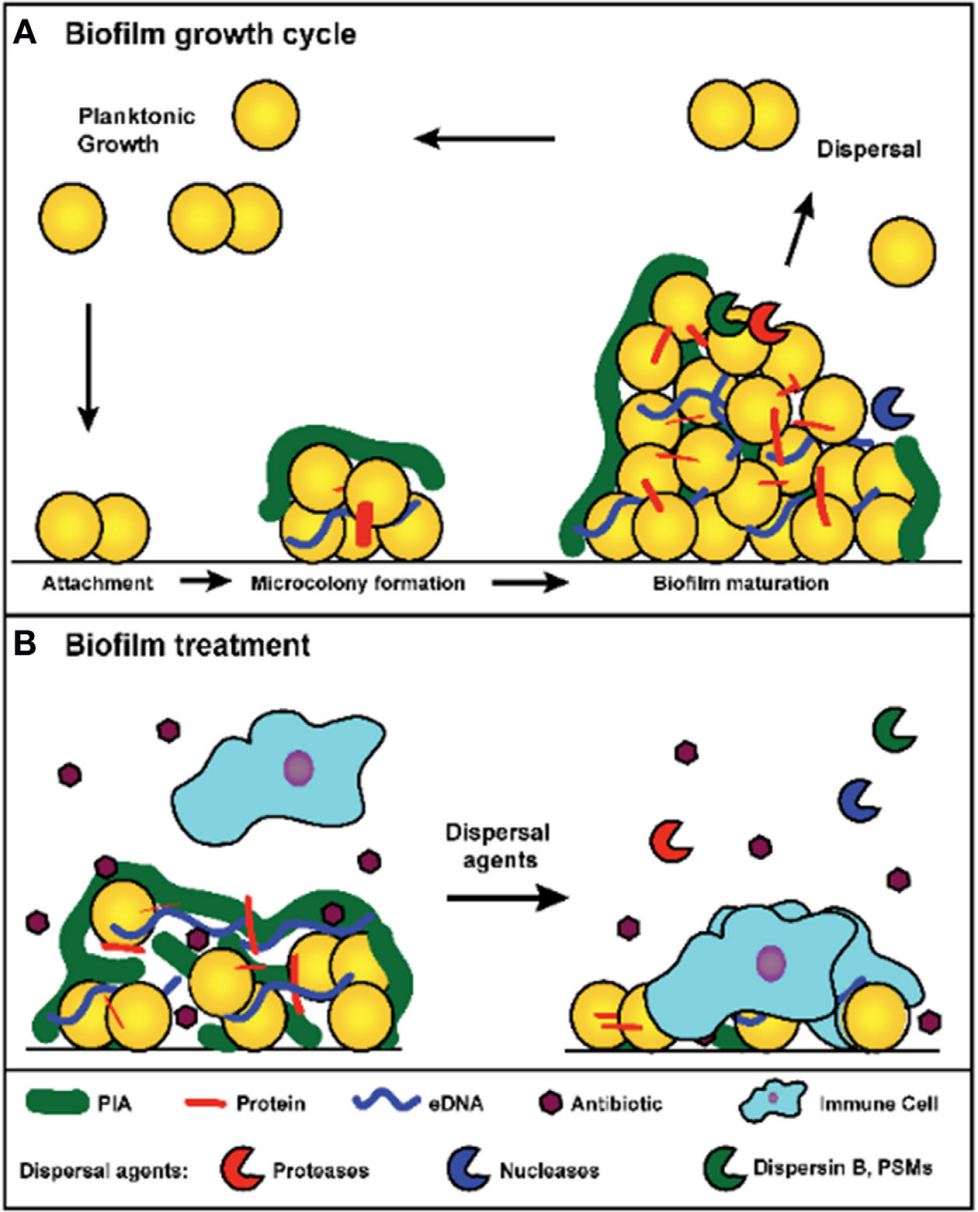
**(A)** Model of *S. aureus* biofilm growth cycle. In summary, upon coming into contact with a surface, planktonic cells attach through surface-associated proteins. Following attachment, cells divide and begin production of the extracellular matrix, which leads to the formation of a microcolony. As cell division continues, biomass accumulates and a mature biofilm is formed. Environmental signals within the biofilm trigger the activation of dispersal mechanisms, and upon dispersal, cells re-enter a planktonic growth state and can seed new sites for biofilm formation. **(B)** Treatment of a *S. aureus* biofilm. Antibiotic exposure will kill susceptible planktonic cells and metabolically active cells near the surface of the biofilm. However, persister cells and metabolically dormant cells within the biofilm survive and remain protected from immune defenses by the biofilm matrix. Treatment with dispersal agents increases the effectiveness of antibiotic penetration and promotes clearance. Antibiotic sensitive cells within the biofilm are exposed and killed after degradation of the matrix, and the antibiotic tolerant cells (such as persisters) survive and are susceptible to the immune system.

A biofilm is defined as a sessile microbial community in which cells are attached to a surface or to other cells and embedded in a protective extracellular polymeric matrix. This mode of growth exhibits altered physiologies with respect to gene expression and protein production (Parsek and Singh, 2003; Archer et al., 2011; Kiedrowski and Horswill, 2011). Biofilm developmental stages have been defined by many and can be divided into at least three major events: initial attachment, biofilm maturation, and dispersal (Figure 1A). During initial attachment, an individual planktonic cell will reversibly associate with a surface, and if the cell does not dissociate, it will bind irreversibly to the surface. Attachment is mediated through surface proteins, referred to as microbial surface components recognizing adhesive matrix molecules (MSCRAMMs) (Foster et al., 2014). During infection, these proteins play major roles in attachment to host factors such as fibrinogen, fibronectin, and collagen. Biofilm maturation occurs through cell division and the production of the extracellular polymeric matrix. The composition of the biofilm matrix varies between strains, but in general can contain host factors, polysaccharide, proteins, and extracellular DNA (eDNA) (Montanaro et al., 2011; Cue et al., 2012; Foster et al., 2014). Following biofilm accumulation, cells within the biofilm can reactivate to a planktonic state through dispersal (Boles and Horswill, 2011). The major mechanisms of *S. aureus* dispersal will be explored in this review.

Growth in a biofilm plays an important role during infection by providing a defense against several clearance mechanisms. The biofilm matrix can impede the access of certain types of immune defenses, such as macrophages, which display incomplete penetration into the biofilm matrix and “frustrated phagocytosis” (Scherr et al., 2014). Additionally, biofilm cells display increased tolerance to antibiotics (de la Fuente-Nunez et al., 2013). In contrast to heritable antibiotic resistance mechanisms, biofilm-associated antibiotic tolerance is a transient state in which normally susceptible bacteria enter an altered physiology that decreases sensitivity. When these cells disperse and reenter a planktonic state, they regain normal antibiotic sensitivity (Singh et al., 2009). One suggested mechanism for this phenomenon is that the biofilm matrix blocks access to actively growing cells within the biofilm by decreasing antibiotic diffusion rates. However, this mechanism is dependent on the type of antibiotic, as certain antibiotics are capable of penetrating the biofilm (Singh et al., 2010). An alternative proposal is that antibiotic tolerance is due to the development of physiologically dormant persister cells that form stochastically during biofilm growth (Lewis, 2010). Due to their decreased metabolic activity, they are inherently resistant to antibiotics. Furthermore, persister cells develop at greater rates within a biofilm than within actively growing planktonic cultures (Singh et al., 2009). As such, they are thought to play a large role in the recalcitrance of biofilm-associated infections.

Beyond offering resistance to clearance mechanisms, biofilms also play an important role in the progression of chronic diseases. Following the establishment of a biofilm, individual cells can disperse from the original biofilm and either seed new sights of infection or mediate an acute infection such as sepsis (Costerton et al., 1999). The role played by the *S. aureus* quorum sensing system during dispersal supports this model (Boles and Horswill, 2008; Lauderdale et al., 2010). Dispersal has been the focus of many recent studies due to its importance in chronic infections and the biofilm model of growth, and an analysis of major dispersal mechanisms has led to the development of dispersal-mediated treatment options for biofilm infections (Kaplan, 2010; Boles and Horswill, 2011). This review discusses the major mechanisms for *S. aureus* biofilm dispersal. In addition, it analyzes the potential for developing dispersal-mediated treatments for biofilm infections (Figure 1B).

## The *Staphylococcus aureus* biofilm matrix

The *S. aureus* biofilm matrix is a complex glue that encases all of the cells in the mature structure, and it is thought to be composed of host factors, secreted and lysis-derived proteins, polysaccharide, and eDNA. The contribution of each of these factors depends heavily on the strain background and on environmental conditions (Fitzpatrick et al., 2005; Abraham and Jefferson, 2012). Furthermore, the effectiveness of many dispersal mechanisms is dependent on the matrix composition (Chaignon et al., 2007; Izano et al., 2008). A brief background on the major components of the biofilm matrix and factors involved in generating these components will be provided.

A major constituent of the biofilm matrix is polysaccharide intercellular adhesin (PIA), also known as polymeric N-acetyl-glucosamine (PNAG) (O'Gara, 2007). PIA is an important component in both *S. aureus* and *S. epidermidis* biofilms that is produced by enzymes encoded in the *icaADBC* locus. PIA is composed of β-1,6-linked *N*-acetylglucosamine polymer, and the proteins encoded in the *ica* locus are responsible for the synthesis, export, and modification of PIA. The PIA polymer plays an important role in the structural integrity of biofilms *in vitro* and *in vivo*, although numerous studies have identified *S. aureus* strains capable of forming *ica*-independent biofilms (Beenken et al., 2003; Fitzpatrick et al., 2005; Toledo-Arana et al., 2005; Lauderdale et al., 2009; Brooks and Jefferson, 2014). The matrix components of these biofilms were later identified as proteins and eDNA (O'Neill et al., 2007, 2008; Rhode et al., 2007; Boles et al., 2010), which function as intercellular adhesins in the absence of PIA.

Many proteins have been implicated as important components in attachment and biofilm matrix development. These include surface-associated proteins such as protein A, fibrinogen-binding proteins (FnBPA and FnBPB), *S. aureus* surface protein (SasG), biofilm-associated protein (Bap), and clumping factor B (ClfB) (Cucarella et al., 2001; Corrigan et al., 2007; O'Neill et al., 2008; Merino et al., 2009; Geoghegan et al., 2010; Abraham and Jefferson, 2012). Many of these factors play a role both in attachment and accumulation. In addition, secreted proteins such as extracellular adherence protein (Eap), and beta toxin (Hlb) play a role in biofilm maturation (Huseby et al., 2010; Sugimoto et al., 2013). However, the importance of individual proteins varies largely between strains (Artini et al., 2013). For example, Bap-dependent biofilms have not been identified in any human isolates, and as such it is more likely that Bap plays a role in bovine mastitis (where it was originally identified) than in human diseases (Lasa and Penades, 2006). In addition to dedicated matrix proteins, intracellular proteins have been identified within the biofilm matrix. These proteins are likely released by cell lysis and nonspecifically incorporated into the matrix (Foulston et al., 2014). The relative importance of lysis-derived proteins is not yet understood.

The most recently identified and appreciated biofilm matrix component is eDNA. Due to the negative charge of the DNA polymer, eDNA potentially acts as an electrostatic polymer that anchors cells to a surface, to host factors, and to each other. Early biofilms are most sensitive to DNase treatment, suggesting that eDNA may be important during attachment (Mann et al., 2009). eDNA is produced through the autolysis of a subpopulation of the biofilm cells (Thomas and Hancock, 2009), and this altruistic suicide is mediated through the activity of murein hydrolases, encoded by the *atl* and *lytM* genes. Murein hydrolases degrade peptidoglycan and typically play an important role during cell wall rearrangements and cell division. Increased expression of these enzymes allows for autolysis in *S. aureus*. Autolysis is regulated through the activity of two operons, *cidABC* and *lrgAB*, that function together in a manner similar to bacteriophage holin/antiholin systems (Sadykov and Bayles, 2012). CidA, the holin in this system, oligomerizes in the cell membrane and results in the formation of a pore that is utilized for the transport of the murein hydrolase. LrgAB functions as the antiholin and prevents the activity of CidA. Studies have indicated that the regulation of autolysis is tied to micro-environmental niches that form within a biofilm, such as the hypoxic conditions found near the base of the biofilm (Moormeier et al., 2013).

There are some reported examples of interactions between eDNA and specific proteins within the biofilm. The best characterized example in *S. aureus* is beta toxin (Huseby et al., 2010), which is a secreted neutral sphingomyelinase capable of lysing erythrocytes and lymphocytes. However, it is structurally related to the DNaseI superfamily of proteins and is able to bind DNA. Beta toxin forms insoluble oligomers upon binding DNA that could serve as a bridge to hold the biofilm structure together. Deletion of the *hlb* gene correlates with a reduction in biofilm formation in both *in vitro* and *in vivo* models. Additional studies have implicated that proteins with non-specific DNA-binding activity may be important matrix components in multiple bacterial species, as antibodies against IHF, a common member of the DNABII family of proteins, are capable of disrupting existing biofilms in *in vitro* and *in vivo* models (Goodman et al., 2011; Novotny et al., 2013).

## Biofilm dispersal mechanisms

The primary biofilm dispersal strategy utilized by *S. aureus* is the production of various exo-enzymes and surfactants to degrade the extracellular polymeric matrix. The effectiveness of individual mechanisms is highly dependent on the matrix composition of the *S. aureus* strain in question (Chaignon et al., 2007; Kiedrowski et al., 2011). In general, mechanisms utilizing the enzymatic self-destruction of either protein and/or eDNA in the matrix are less effective at dispersing polysaccharide-dependent biofilms. In contrast, the mechanisms specifically targeting PIA are ineffective against polysaccharide-independent biofilms. In this review, the dispersal mechanisms targeting each matrix component will be discussed, with an emphasis on self-targeting enzymatic mechanisms (Table 1), and two recently described fundamental processes with biofilm dispersing activity will also be covered. Non-specific mechanisms, such as the surfactant activity of phenol-soluble modulins (PSMs), are effective against most *S. aureus* biofilms and are reviewed elsewhere (Peschel and Otto, 2013).

**Table 1:** **Biofilm dispersal mechanisms**.

**Dispersal agent**	**Mechanism**	**References**	**Specific factor**
Proteases	Degradation of proteinaceous matrix components	McGavin et al., 1997; O'Neill et al., 2008	V8 protease (SspA)
		Mootz et al., 2013	Staphopains (Cysteine Proteases)
		Abraham and Jefferson, 2012	Aureolysin (Aur)
		Marti et al., 2010	Aur, SspA
		Lauderdale et al., 2010; Shukla and Rao, 2013	Proteinase K
		Beenken et al., 2003; Trotonda et al., 2005; Tsang et al., 2008; Zielinska et al., 2012	*sarA* regulation
		Lauderdale et al., 2009	*sigB, agr* regulation
agr activation by AIP	Expression of agr regulated factors (proteases and PSMs)	Yarwood et al., 2004; Boles and Horswill, 2008; Lauderdale et al., 2010	AIP
Phenol-soluble modulins	Surfactant-mediated dispersal	Peschel and Otto, 2013	PSMs
*S. epidermidis* Esp	Degradation of proteinaceous matrix components; inhibition of autolysis through Atl degradation	Iwase et al., 2010; Chen et al., 2013; Sugimoto et al., 2013	Esp
Nucleases	Degradation of eDNA	Kiedrowski et al., 2011	Nuc
		Kiedrowski et al., 2014	Nuc2
Dispersin B	Degradation of polysaccharide matrix components	Kaplan et al., 2004; Donelli et al., 2007	DisB
D-amino acids	Protein synthesis inhibition in *B. subtilis*, unknown in *S. aureus*	Kolodkin-Gal et al., 2010; Hochbaum et al., 2011; Leiman et al., 2013; Sanchez et al., 2013	D-amino acids
Stringent response inhibition	Unknown	de la Fuente-Nunez et al., 2014; Reffuveille et al., 2014	Peptide 1018

### Enzymatic dispersal mechanisms

#### Protease-mediated dispersal

*S. aureus* produces 10 secreted proteases, including seven serine proteases (SspA and SplA-F), two cysteine proteases (SspB and ScpA), and one metalloprotease (Aur) (Shaw et al., 2004). The role of proteases in biofilm dispersal was initially characterized during the analysis of *S. aureus* strains deficient in the global regulators *sarA* and *sigB* (Bronner et al., 2004) that were unable to form biofilm (Beenken et al., 2003; Trotonda et al., 2005; O'Neill et al., 2008). Characterization of these mutants revealed that the observed biofilm phenotypes resulted from elevated protease activity levels (Tsang et al., 2008; Lauderdale et al., 2009; Marti et al., 2010; Zielinska et al., 2012; Mootz et al., 2013). The high protease activity results in the degradation of important matrix proteins and destabilization of the biofilm (Zielinska et al., 2012). This phenotype could be reversed by the deletion of multiple protease genes or the addition of protease inhibitors (McGavin et al., 1997; Tsang et al., 2008; Mootz et al., 2013). The ability of the V8 serine protease (SspA), the staphopains (SspB and ScpA), and aureolysin (Aur) to disrupt biofilms have been demonstrated (Table 1), with the relative importance of each varying between strains and conditions. The V8 serine protease can degrade FnBPs and Bap (McGavin et al., 1997; O'Neill et al., 2008; Marti et al., 2010), and aureolysin can degrade ClfB and Bap to mediate biofilm disruption (Marti et al., 2010; Abraham and Jefferson, 2012). While the staphopains can disrupt the biofilm matrix, no target proteins have yet been characterized (Mootz et al., 2013). Additional targets such as Atl, Spa, and SasG have been proposed, but have not been linked to individual proteases (Lauderdale et al., 2009; Kolar et al., 2013). Despite the identification of some specific matrix proteins as targets for degradation, the large number of proteases and potential matrix protein targets will require proteomic analysis to dissect the complex mechanism behind protease-mediated dispersal.

The production of proteases is positively regulated through the *S. aureus* quorum sensing system, *agr* (Thoendel et al., 2011). The *agr* system is activated upon detection of an autoinducing peptide (AIP) that is encoded and produced by the *agr* operon. The AIP is detected by a two-component system that regulates virulence through the production of a regulatory RNA, RNAIII. The *agr* system regulates the virulence state of the cell by activating the production of secreted toxins and enzymes and the down-regulation of surface factors. The *agr* system induces the expression of both proteases and PSMs, which act as surfactants to disperse biofilms (Peschel and Otto, 2013). Thus, activation of the *agr* system can result in a shift from a biofilm state to a planktonic state of growth. This has been demonstrated through the addition of AIP to existing biofilms, which results in complete dispersal (Boles and Horswill, 2008; Lauderdale et al., 2010), and through the use of fluorescent reporters, which demonstrated that cells detach from the biofilm after *agr* activation (Yarwood et al., 2004).

In addition to native *S. aureus* proteases, recent studies have indicated that the production of non-native proteases may impact *S. aureus* biofilm growth in bacterial communities. The serine protease Esp produced by *S. epidermidis* has been shown to disperse *S. aureus* biofilms (Sugimoto et al., 2013). This was first identified when it was observed that *S. aureus* colonization rates of the human nares negatively correlate with colonization rates of *esp* positive *S. epidermidis* (Iwase et al., 2010). Following this discovery, it was shown that Esp is able to cleave an array of *S. aureus* proteins, including Eap, FnBPA, and Atl (Chen et al., 2013; Sugimoto et al., 2013). The mechanism of Esp-mediated dispersal is thus two-fold: Esp degrades matrix proteins important for intercellular adhesion and prevents the release of eDNA by degrading murein hydrolase.

#### Nuclease-mediated dispersal

*S. aureus* produces two extracellular nucleases, referred to here as nuclease (Nuc) and nuclease2 (Nuc2) (Tang et al., 2008). The production of the major secreted Staphylococcal nuclease, also known as micrococcal nuclease or thermonuclease, is conserved across most clinical isolates and is produced *in vivo*. A recent study utilized this fact and developed a nuclease-specific probe for imaging *S. aureus* infections (Hernandez et al., 2014). Nuclease is regulated by the global regulator *sigB* and the SaeRS two-component system (Kiedrowski et al., 2011; Olson et al., 2013), and the expression of *nuc* is greatly reduced during biofilm growth conditions, suggesting that Nuc may play a role in the biofilm growth cycle (Olson et al., 2013).

Two major roles have been proposed for Nuc during infection, the disruption of neutrophil extracellular traps (NETs) and modulating biofilm development. It has been shown that the expression of nuclease results in reduced biofilm formation *in vitro*, while a *nuc* mutant displays enhanced biofilm formation (Mann et al., 2009; Kiedrowski et al., 2011). These phenotypes correlate with levels of eDNA accumulation during biofilm growth, where lack of nuclease results in the preservation of high molecular weight eDNA (Mann et al., 2009; Kiedrowski et al., 2011). This agrees with an earlier study that found a minimum size of 11 kb fragments was necessary for biofilm integrity (Izano et al., 2008). The second role proposed for nuclease during infection is the evasion of NETs. NETs are a newly discovered killing mechanism utilized by neutrophils against bacterial infections. Activated neutrophils secrete nuclear DNA at the site of infection to entrap bacteria and enhance bacterial killing. Nuclease is able to degrade NETs and promote resistance against killing by neutrophils (Berends et al., 2010; Thammavongsa et al., 2013). The relative importance of each activity during infection has not yet been explored. Overall, *in vivo* studies indicate that *nuc* mutants are attenuated during infection (Berends et al., 2010; Olson et al., 2013). However, it is unclear whether this attenuation results from a reduced ability to disperse from a biofilm and disseminate to new sites, an increased susceptibility to killing by neutrophils, or the inability to scavenge nucleotides in the host. It is possible that all these functions of nuclease are important during infection.

In contrast to nuclease, the function of Nuc2 is still relatively unknown. This is in part due to the difficulty in studying Nuc2 in wild type backgrounds, as its activity is masked by Nuc. A recent study has shown that Nuc2 is a membrane-bound nuclease with an extracellular catalytic domain. Nuc2 activity is detectable in a *nuc* mutant, but the activity is very low (Kiedrowski et al., 2014). This is likely due to low expression levels, as mechanistic studies demonstrated that the Nuc2 catalytic domain is functional. Addition of purified Nuc2 was able to partially disperse existing biofilms, suggesting that Nuc2 could play a role in localized dispersal during infection. This localized dispersal could result in the formation of channels within the biofilm or supplement Nuc activity in high flow environments (such as those seen during endocarditis) where Nuc would be unable to accumulate. However, further studies will be necessary to determine the function of Nuc2 *in vivo*.

#### Dispersin B-mediated dispersal

The enzyme dispersin B isolated from *Actinobacillus actinomycetemcomitans* is able to disperse polysaccharide-dependent *Staphylococcus epidermidis* and *S. aureus* biofilms (Kaplan et al., 2004). Dispersin B disrupts the biofilm by hydrolyzing the glycosidic linkages of PIA. No homolog of dispersin B has been identified in the *S. aureus* genome so it is unlikely the organism utilizes this mechanism for dispersal during biofilm growth. However, treatment of biofilms with dispersin B does result in increased susceptibility to antimicrobials (Donelli et al., 2007). Thus, dispersin B could be developed as a potential anti-biofilm treatment.

### Broad-spectrum dispersal mechanisms

#### D-amino acids

It has been reported that D-amino acids produced during late stationary phase induce biofilm dispersal in multiple bacteria, including *S. aureus* (Kolodkin-Gal et al., 2010). The role of D-amino acids in dispersal was initially discovered in *Bacillus subtilis*. The proposed mechanism behind this dispersal was the incorporation of D-amino acids into the peptidoglycan, resulting in a failure to attach the major matrix protein, TasA, to the cell wall. Subsequently, this resulted in decreased intercellular adhesion via the detachment of existing TasA fibers. This dispersal mechanism was tested in additional bacterial species, including *S. aureus* and *P. aeruginosa*, where a similar phenotype were observed (Hochbaum et al., 2011).

However, a recent study has found that the effect of D-amino acids observed in *B. subtilis* was due to a strain specific mutation in the *dtd* gene (Leiman et al., 2013). *dtd* encodes a D-tyrosyl-tRNA deacylase and is responsible for preventing the misincorporation of D-amino acids into protein. As such, the D-amino acid biofilm dispersal effect observed in the *dtd* mutant was due to a growth defect caused by interference with protein synthesis. The impact of D-amino acids on *S. aureus* biofilm is therefore unclear and requires further investigation. However, D-amino acids may still offer clinical applications for the prevention of biofilm infections. It has been shown that pre-treatment of polymeric surfaces with D-amino acids reduces *S. aureus* biofilm formation *in vitro* (Hochbaum et al., 2011; Sanchez et al., 2013).

#### Stringent response

The stringent response is a general bacterial system triggered by nutrient starvation that allows cells to adapt to stressful conditions, such as those seen during infection (Srivatsan and Wang, 2008). During nutrient starvation, the alarmone ppGpp is produced by RelA/SpoT homologs and elicits regulatory changes that switch the cell to a metabolically inactive state. Studies have linked the stringent response to virulence and biofilm formation in multiple bacterial species (Lemos et al., 2004; Nguyen et al., 2011; Vogt et al., 2011; Chavez de Paz et al., 2012; He et al., 2012; Wexselblatt et al., 2012; Sugisaki et al., 2013). In *S. aureus*, evidence suggests the stringent response plays a role during infection (Geiger et al., 2010), but its impact on biofilm has not been extensively studied.

A recent study identified a synthetic cationic peptide capable of dispersing biofilms in a large number of clinically relevant bacterial pathogens, including *S. aureus*, without inhibiting planktonic growth (de la Fuente-Nunez et al., 2014). The peptide affected both Gram-negative and Gram-positive organisms, implicating that the peptide was targeting a general bacterial process. Further investigation determined that the peptide was inhibiting the stringent response through a direct interaction with ppGpp that resulted in the degradation of the alarmone. This result indicates that the metabolic state of the cell plays some role in dispersal. Additional research will be necessary to explore the role of stringent response in *S. aureus* biofilm dispersal.

## Implications for clinical treatment of biofilm infections

Biofilm dispersal has drawn interest as a potential means of treating persistent *S. aureus* infections. The intentional dispersal of a biofilm coupled with antibiotic therapy would expose and kill metabolically active cells and render any remaining persister cells vulnerable to the immune system (Figure 1B). Increased antibiotic susceptibility has been observed with most dispersal agents, including many industrially produced enzymes such as dispersin B, proteinase K, and DNaseI (Lauderdale et al., 2010; Kaplan et al., 2012b; Shukla and Rao, 2013; Reffuveille et al., 2014). The efficacy of dispersal-mediated treatments could potentially be improved by the inclusion of a drug targeting persister cells (Conlon et al., 2013). In addition to the treatment of existing infections, dispersal mechanisms could be utilized in the prevention of biofilm formation associated with implanted medical devices. Several studies have found that pretreatment of polymeric surfaces with dispersing agents can reduce biofilm formation *in vivo* (Donelli et al., 2007; Sanchez et al., 2013). The slow release of dispersal agents from the implanted device should prevent biofilm accumulation and facilitate clearance of the bacteria by the immune system. While these approaches sound promising, there are several concerns that have not yet been thoroughly addressed. First, induced dispersal could result in acute infections if the antibiotic fails to eradicate the released cells. Sub-inhibitory concentrations of certain antibiotics have been linked to enhanced *agr* activation (Joo et al., 2010), which could accelerate an acute response. Sub-inhibitory concentrations of β-lactams have also been linked to the induction of eDNA release and biofilm formation (Kaplan et al., 2012a), which could be counter-productive when coupled with a dispersal agent. Embolisms resulting from the release of cell clumps embedded in matrix components represent another major concern. Studies will need to address these challenges before dispersal agents are tested in a clinical setting.

## Concluding remarks and future perspectives

The ability to form a biofilm is an important virulence determinant for the persistence of *S. aureus* chronic infections. In this review, we focused on the strategies utilized by *S. aureus* to escape from a biofilm through dispersal and disseminate to other body sites. Ongoing research continues to improve our understanding of the exo-enzymes and surfactants that degrade the biofilm matrix and release cells into the surrounding environment. The enzymes that have drawn the most attention are the secreted cysteine proteases (staphopains), V8 serine protease (SspA), and nuclease (Nuc). The relative importance of each enzyme will depend on the strain-specific composition of the biofilm matrix. The proteases and surfactant molecules are under *agr* quorum-sensing control, and activation of this regulatory system is a known dispersing mechanism.

Going forward, additional studies are necessary to fill specific knowledge gaps. The targets of the major proteases (V8, Aur, staphopains) are still not fully described, although some candidate surface proteins, like the FnBPs and ClfB, have been identified. The function of Nuc in biofilm dispersal has not been examined in detail. It is likely other exo-enzymes, such as hyaluronidase and lipases, are also important in dispersal mechanisms, but have not been fully investigated in biofilm studies (Rosenthal et al., 2014). In addition to the matrix-degrading mechanisms, it is possible that D-amino acids and the stringent response may play a role in dispersal, but further work is needed to better characterize these mechanisms. Perhaps the area of greatest need is confirming dispersal mechanisms in relevant animal models of infection and testing the efficacy of dispersal agents in treating biofilm infections. Additionally, coupling these agents with antibiotic therapy to facilitate clearance of a recalcitrant infection has received little attention. Overall our knowledge of enzymatic dispersal mechanisms has expanded in recent years, but many details still remain unclear. Further work on the topic will allow for the development of better treatment options for biofilm-mediated diseases.

### Conflict of interest statement

The authors declare that the research was conducted in the absence of any commercial or financial relationships that could be construed as a potential conflict of interest.
